# Gender Differences in the Association Between Anxiety and Interoceptive Insight

**DOI:** 10.1111/ejn.16672

**Published:** 2025-01-13

**Authors:** Olivia K. Harrison, Laura Köchli, Stephanie Marino, Lucy Marlow, Sarah L. Finnegan, Ben Ainsworth, Benjamin J. Talks, Bruce R. Russell, Samuel J. Harrison, Kyle T. S. Pattinson, Stephen M. Fleming, Klaas E. Stephan

**Affiliations:** ^1^ Department of Psychology University of Otago Dunedin New Zealand; ^2^ Nuffield Department of Clinical Neurosciences University of Oxford Oxford UK; ^3^ Translational Neuromodeling Unit University of Zurich and ETH Zurich Zurich Switzerland; ^4^ Department of Psychology University of Bath Bath UK; ^5^ Population Health Sciences Institute Newcastle University Newcastle Upon Tyne UK; ^6^ Birmingham Medical School Birmingham Medical Research Expeditionary Society Birmingham UK; ^7^ School of Pharmacy University of Otago Dunedin New Zealand; ^8^ Department of Experimental Psychology University College London London UK; ^9^ Wellcome Centre for Human Neuroimaging University College London London UK

**Keywords:** breathing, computational psychiatry, insight, interoception, metacognition

## Abstract

Anxiety is one of the most common and debilitating mental health disorders, and is related to changes in interoception (perception of bodily states). While anxiety is more prevalent in women than men, gender differences in interoception‐anxiety associations are often overlooked. Here, we examined gender‐specific relationships between anxiety and interoception in the breathing domain, utilising multicentre data pooled from four study sites (*N* = 175; 51% women). State anxiety scores were quantified via the Spielberger State–Trait Anxiety Inventory, and breathing‐related interoceptive dimensions via an inspiratory load task to quantify sensitivity, decision bias, metacognitive bias (confidence in interoceptive decisions), and metacognitive insight (congruency between performance and confidence). Regression analyses revealed a significant negative relationship between state anxiety and metacognitive bias (β = −0.28; *p* = 0.01) and insight (β = −0.09; 95% highest density interval [HDI] in a hierarchical Bayesian regression = [−0.18, −0.004]) across the whole sample, while state anxiety did not relate to interoceptive sensitivity nor decision bias. While no mean interoceptive effects relating to gender were observed, the relationship between anxiety and metacognitive insight towards breathing was driven by women (women: β = −0.18; HDI = [−0.31, −0.05]; men: β = 0.02; HDI = [−0.12, 0.15]) with a significant interaction effect (β difference = −0.20; HDI = [−0.37, −0.01]), which did not hold for trait anxiety nor depression measures. In summary, state anxiety was associated with decreased metacognitive bias across all participants, while decreased interoceptive insight was only associated with anxiety in women but not men. Therefore, treatment programmes focusing on interoceptive metacognitive bias may be useful for all anxiety patients, while interoceptive insight might represent a specific treatment target for women with anxiety.

## Graphical abstract

We found state anxiety to be related to both the metacognitive bias and insight domains of respiratory interoception. However, the relationship between state anxiety and interoceptive insight was only present in women and not men, while metacognitive bias was strongest in women and trending in men, with no difference between genders. Figure created in BioRender. Harrison, O. (2024) https://BioRender.com/e81j572.

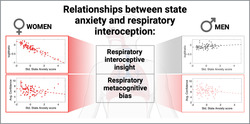

**Abbreviations**
FDT: filter detection task
GLM: general linear model
HDI: highest density interval
HMeta‐d: hierarchical metacognitive d‐prime estimation
JAGS: Just Another Gibbs Sampler
MCMC: Markov chain Monte Carlo
RHMeta‐d: regression hierarchical metacognitive d‐prime estimation
UK: United Kingdom

## 1 Introduction

Anxiety is one of the most common mental health disorders that is often associated with debilitating somatic symptoms (e.g., racing heart or shortness of breath). Recent theories propose a key role for interoception (the perception of bodily states) in conditions such as anxiety (Paulus and Stein 2010; Quadt, Critchley, and Garfinkel 2018). Interoception is a critical part of closed‐loop interactions between the brain and body, maintaining a continuously updating representation of body state across both conscious and subconscious levels (Quadt, Critchley, and Garfinkel 2018; Khalsa et al. 2017; Petzschner et al. 2021; Murphy 2023; Craig 2002; Toussaint, Heinzle, and Stephan 2024). Interoception encompasses multiple layers of processing (Greenwood and Garfinkel 2024), and lower‐level measures such as objective performance in perceptual detection tasks (i.e., sensitivity towards stimulus detection) can be differentiated from more ‘metacognitive’ and higher‐order awareness dimensions, where metacognition refers to the ability to accurately reflect and monitor cognitive or perceptual processes (Quadt, Critchley, and Garfinkel 2018; Maniscalco and Lau 2012; Garfinkel et al. 2015).

Respiratory symptoms are a core feature of anxiety (Masaoka and Homma 2001; Homma and Masaoka 2008). While elevated autonomic arousal linked to anxiety is often associated with marked increases in ventilation (Dampney 2015; Dampney et al. 2013; Kluger, Gross, and Keitel 2024), periods of apnoea (breath holds) can also occur and are thought to be (at least in part) driven by inhibitory connections from the central nucleus of the amygdala to respiratory brainstem centres (Feinstein, Gould, and Khalsa 2022). Importantly, the subjective perception of these breathing symptoms (and their downstream physiological effects) can be highly frightening, fuelling further anxiety (Paulus 2013; Leivseth et al. 2012). While breathing‐related interoceptive research remains relatively novel compared to work in the cardiac domain (Domschke et al. 2010; Adams et al. 2022), findings have demonstrated pervasive interoceptive dysfunction with elevated anxiety, including decreased sensitivity (Tiller, Pain, and Biddle 1987; Garfinkel et al. 2016; Harrison, Köchli et al. 2021), decreased metacognitive bias (measured via self‐reported confidence) (Harrison, Köchli et al. 2021; Harrison, Marlow et al. 2021) and decreased metacognitive insight (congruency between performance and confidence in interoceptive judgements) (Harrison, Marlow et al. 2021). However, the reported pattern of interoceptive dysfunction has been inconsistent across studies, likely driven by small sample sizes, demographic differences and utilising measurements of different anxiety constructs.

Anxiety can present in both state (i.e., the psychophysiological state experienced in the moment) and trait (i.e., more stable individual tendencies to experience periods of anxiety) forms (Spielberger 2010; Leal et al. 2017). Beyond their initial conceptualisation as a unidimensional construct (Spielberger 2010) (where greater trait anxiety predisposes individuals to experience greater state anxiety), state and trait anxiety have been shown to differentially relate to brain structure and function (Saviola et al. 2020), and diverge in response to distinctive types of threat. Specifically, while interpersonal threat generates experiences of state anxiety that are closely related to trait anxiety scores, the presence of a physical or bodily threat generates state anxiety that is unrelated to trait anxiety (Leal et al. 2017). Therefore, state and trait anxiety may plausibly differ in their relationship with interoception, with state anxiety potentially more tightly linked with changes in bodily symptoms.

While anxiety is approximately twice as prevalent in women than men (Steel et al. 2014), the causes of this difference are not well understood. It has been hypothesised that underlying differences in interoception may contribute to these disparities (Murphy, Viding, and Bird 2019), with previous work demonstrating that women present with greater subjective interoceptive awareness (Grabauskaitė, Baranauskas, and Griškova‐Bulanova 2017), higher somatic complaints (Haug, Mykletun, and Dahl 2004; Barsky, Peekna, and Borus 2001), yet reduced objective interoceptive sensitivity in both respiratory (Harver, Katkin, and Bloch 1993) and cardiac domains (Grabauskaitė, Baranauskas, and Griškova‐Bulanova 2017; Harver, Katkin, and Bloch 1993; Murphy et al. 2018). Therefore, in this study, we investigated whether gender differences exist in the relationship between both state and trait anxiety and any of the dimensions of respiratory‐based interoception. We also considered the relationship between interoception and depressive symptoms, which are highly co‐morbid with anxiety. Using a task focused on interoception of breathing via detection of very small variations in inspiratory load, we quantified perceptual sensitivity, decision bias, metacognitive bias (self‐reported confidence) and metacognitive insight into breathing perception. By pooling data across multiple study sites, we were able to investigate discrepancies in the relationships reported by previous literature using smaller study samples (Harrison, Köchli et al. 2021; Harrison, Marlow et al. 2021), and investigate any potential interoceptive differences between men and women.

## 2 Methods

To systematically evaluate aspects of breathing‐related interoception, we utilised data from healthy volunteers from four study sites in Zurich, Switzerland (*N* = 78; Ethics BASEC‐No. 2017–02330) (Harrison, Köchli et al. 2021), Oxford, UK (*N* = 30; Ethics 17/EM/0107) (Harrison, Marlow et al. 2021), Bath, UK (*N* = 30; Ethics 19–119; unpublished data) and Birmingham, UK (*N* = 37; Ethics R60699/RE001) (Talks et al. 2022). In total, this resulted in a sample size of *N* = 175 with 51% women (ethical approval for data pooling provided by the Zurich Cantonal Ethics Committee, BASEC‐No 2020–00645). While state anxiety was our pre‐specified primary variable of interest, both state and trait anxiety scores were measured using the Spielberger State–Trait Anxiety Inventory (STAI‐S and STAI‐T) (Spielberger 2010) and depression was measured using the Centre for Epidemiological Studies Depression Scale (CES‐D) (Radloff 1977) (available data: STAI‐S *n* = 175; STAI‐T *n* = 173; CES‐D *n* = 159). Questionnaires were collected immediately prior to the breathing‐related interoceptive data (detailed below).

**FIGURE 1:**
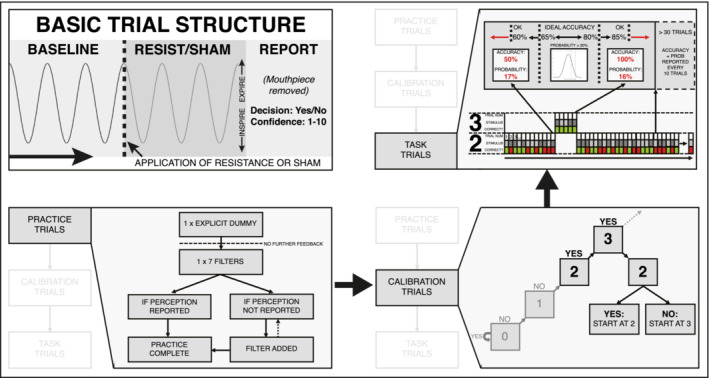
Visualisation of the task structure and performance algorithm. Top left: Overview of the basic trial structure for the task. Participants take three normal size/pace breaths (with the sham filter attached), and during the third exhalation (indicated by the participant raising their hand and the dotted line), the experimenter either swaps the sham for a number of stacked filters (to provide a very small inspiratory resistance) or removes and replaces the sham filter. Following three more breaths, the participant removes the mouthpiece and reports whether they thought it a resistance was added (‘Yes’) or not (‘No’), and how confident they are in their decision on any scale (here 1–10 used, with 1 = *guessing* and 10 = *maximally confident in their decision*). Bottom left: Practice trials, consisting of an explicit dummy (where the participants are told a sham resistance is added), followed by a large load (7 filters) where no feedback is given (this omission of feedback is then maintained for the rest of the experiment). If no resistance is perceived on this trial, filters are added until a correct resistance is reported. Bottom right: Calibration trials, where (starting from the dummy), filters are added until two consecutive resistances are reported Following this, one final calibration trial is performed with one less filter, to determine the starting value for the task trials. Top right: Task trials, where cumulative task accuracy at each trial is transformed (using a beta distribution) into the distribution of underlying accuracies that could have produced the task performance. An upper bound (here 85%) and a lower bound (here 60%) is used to calculate the probability that the participant is performing at the targeted accuracy. If this probability falls below the error risk threshold (here 20%), a filter change is prompted—either the addition of a filter if the accuracy is too low, or the removal of a filter if the accuracy is too high. This continues until either a specified number of trials (here 60 trials) are completed at one filter number. The online algorithm is stopped at 30 trials, and subsequent experimenter intervention can occur every 10 trials if task performance is drastically altered and no longer deemed acceptable. Figures are adapted from Harrison, Garfinkel et al. (2021) under a CCBY licence.

Breathing‐related interoceptive measures were quantified using the Filter Detection Task (FDT) (Harrison, Garfinkel et al. 2021) (Figure 1). This task uses a simple breathing circuit and requires participants to decide whether a very small inspiratory load is added within each trial, as well as indicating their confidence on a scale of 1–10 or 0–100 (1 or 0 = *not at all confident in decision*, 10 or 100 = *extremely confident in decision*). Any confidence scores collected on the 0–100 scale were down‐sampled into 10‐bin intervals for consistency. These inspiratory resistances were designed to be barely perceptible (< 0.48 cm H_2_O/L s^−1^), and overcoming these loads requires a marginally greater inspiratory muscular effort. An adapted staircase algorithm was utilised to calibrate task difficulty and target the perceptual threshold of each participant, whereby their accuracy was significantly above chance level (50%), but not yet fully saturated to 100%. Therefore, task difficulty was altered online via adjustment of the magnitude of inspiratory load until participants were between the tolerance range of 60%–85% accuracy, and 60 trials were completed at this threshold (Figure 1). As 60 trials were required to be completed at each participant's perceptual threshold for further analyses, the tolerance range was chosen to minimise the overall number of trials and thus participant burden of this breathing task.

To calculate the interoceptive measures, breathing‐related interoceptive sensitivity (i.e., perceptual threshold) was taken as the number of filters required for task performance to remain between 60%–85% accuracy, while metacognitive bias was taken as the average in confidence scores across threshold trials. Decision bias and metacognitive insight were calculated using a hierarchical Bayesian model (HMeta‐d) (Fleming 2017), implemented in MATLAB (2020b) and Markov Chain Monte Carlo (MCMC) sampling conducted using JAGS v3.4.0. The model utilises signal detection theory to provide single subject parameter estimates for task difficulty (d′) and decision bias (*c*), where negative *c* values indicate a bias towards reporting ‘yes’ (over‐reporting the presence of a resistance) while positive *c* values denote a bias towards reporting ‘no’ (under‐reporting). Metacognitive insight was assessed by the parameter meta‐d′ (Maniscalco and Lau 2012) from the hierarchical HMeta‐d model. Importantly, Meta‐d′ values were normalised by subject‐specific d′ estimates (thus accounting for any residual differences in accuracy for each participant), resulting in Mratio (meta‐d′/d′) estimates that are independent of task performance, and the logarithm of this metric (logMratio) was taken to meet Gaussian assumptions.

State anxiety, trait anxiety and depression were regressed against each of the interoceptive measures independently. While state anxiety was our primary measure of interest, as pre‐specified by our analysis plan, we post hoc included trait anxiety and depression in our analyses for comparison, as suggested during the review process. Within each of these analyses, a first GLM included a global intercept, anxiety/depression score (*Z*‐scored), and study site regressors. To test the effects of gender, a second GLM was created that included an additional gender regressor and an interaction regressor between anxiety/depression score and gender. Additionally, an alternative (mathematically equivalent) GLM was used to calculate the significance of the anxiety/depression slope within each gender, where the anxiety/depression and interaction regressors were replaced by separate gender‐specific anxiety/depression regressors. These GLMs were used with ordinary least‐squares regression to explain measures of interoceptive sensitivity, decision bias and metacognitive bias, with *p* < 0.05 denoting significance (two‐tailed). Concerning metacognitive insight, we utilised an extension of the HMeta‐d model [RHMeta‐d (Harrison, Garfinkel et al. 2021)], which allows for simultaneous hierarchical estimation of the regression parameters in relation to single‐subject variations in logMratio. To calculate significance for the hierarchically estimated values, the 95% highest density interval (HDI) of the sampled posterior densities were calculated and examined whether they excluded zero (Fleming 2017). Notably, as participants were asked to self‐report their gender in each study (rather than biological sex testing), we have referred to this variable as ‘gender’ throughout. However, by utilising this methodology we note that inference drawn in the present study may differ to other investigations considering biological sex.

Finally, a sensitivity analysis was conducted on the primary state anxiety findings, whereby regression analyses were repeated while omitting the additional study‐specific regressors. This analysis allowed us to examine the extent and influence of the heterogeneity across study sites, by comparing the sensitivity of the results to changes in the model inputs when collapsing across these categorical variables (Nahhas 2024). Comparisons for the reduced model regression estimates to the original model were considered for both quantitative differences (i.e., magnitude differences in regression estimates that are in the same direction) as well as qualitative differences (i.e., regression estimates that change in direction or meaningfully affect the magnitude to drastically alter the significance of the findings). All analyses were pre‐specified and any extensions documented in a time‐stamped analysis plan: https://gitlab.ethz.ch/tnu/analysis‐plans/harrison_breathing_anxiety. All code is publicly available: https://github.com/IMAGEotago/BRIMA.

## 3 Results

**FIGURE 2:**
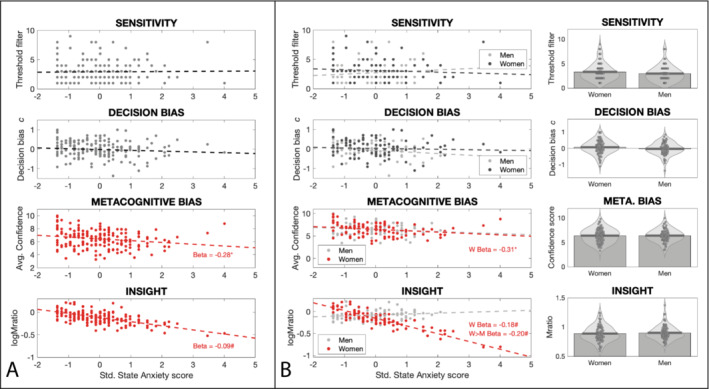
(A) Regression analyses of each of the four interoceptive measures against state anxiety for the whole participant group. Both metacognitive bias and insight demonstrated a significant negative relationship with anxiety, with no effects observed for interoceptive sensitivity nor decision bias: State anxiety slope parameter for metacognitive bias, *p* = 0.01; state anxiety slope parameter for metacognitive insight, HDI [−0.18, −0.004]. (B) Left: Regression analyses of each of the four interoceptive measures against state anxiety for men and women separately: State anxiety slope parameter for metacognitive bias in women, *p* = 0.03; state anxiety slope parameter for metacognitive insight in women, HDI [−0.31, −0.05]; state anxiety slope parameter for metacognitive insight interaction (women > men), HDI [−0.37, −0.01]. Right: No mean differences were observed according to gender for any of the breathing interoceptive measures. *Significant result using ordinary least squares regression with *p* < 0.05. #Significant result using a hierarchical regression model with highest density interval excluding zero.

Table 1 presents the relationships between each interoceptive measure and state anxiety, trait anxiety and depression, both independent of and contingent on gender. Both metacognitive bias and insight into breathing were significantly related to state anxiety across all participants, where greater state anxiety levels were associated with reduced overall confidence in interoceptive decisions, and reduced insight into interoceptive performance (Figure 2A). None of the interoceptive measures demonstrated any mean effect of gender (i.e., non‐significant gender differences; Table 1). Additionally, while there was no effect of gender on the relationship between metacognitive bias and state anxiety (difference in slope estimates between men and women = 0.08; *p* = 0.72), decreased interoceptive insight was only related to greater levels of state anxiety in women but not men (women: β = −0.18; HDI = [−0.31, −0.05]; men: β = 0.02; HDI = [−0.12, 0.15]; interaction women > men = −0.20; HDI of the interaction = [−0.37, −0.01]; Table 1 and Figure 2B).

**TABLE 1:** Regression analyses for each interoceptive measure against state anxiety, trait anxiety and depression as a whole group (left columns) and accounting for gender (right columns).

Interoceptive measure	State anxiety regression		State anxiety and gender regression				
	Intercept	Anxiety slope	Intercept (men)	Gender effect (W > M)	Men: anxiety slope	Women: anxiety slope	Slope interaction
Sensitivity	**β = 2.91**	β = 0.03	**β = 2.72**	β = 0.38	β = 0.24	β = −0.14	β = −0.38
	***p* < 0.01**	*p* = 0.83	***p* < 0.01**	*p* = 0.12	*p* = 0.24	*p* = 0.39	*p* = 0.13
Decision bias	β = −0.02	β = −0.04	β = −0.07	β = 0.10	β = −0.08	β = −0.02	β = 0.06
	*p* = 0.60	*p* = 0.20	*p* = 0.12	*p* = 0.11	*p* = 0.09	*p* = 0.57	*p* = 0.32
Metacognitive bias	**β = 6.44**	**β = −0.28**	**β = 6.42**	β = 0.04	β = −0.23	**β = −0.31**	β = −0.08
	***p* < 0.01**	***p* = 0.01**	***p* < 0.01**	*p* = 0.87	*p* = 0.17	***p* = 0.03**	*p* = 0.72
Insight	**β = −0.12**	**β = −0.09**	β = −0.09	β = −0.07	β = 0.02	**β = −0.18**	**β = −0.20**
	**HDI**	**HDI**	HDI	HDI	HDI	**HDI**	**HDI**
	**[−0.22, −0.03]**	**[−0.18, <−0.01]**	[−0.21, 0.03]	[−0.24, 0.10]	[−0.12, 0.15]	**[−0.31, −0.05]**	**[−0.37, −0.01]**

Interoceptive measure	Trait anxiety regression		Trait anxiety and gender regression				
	Intercept	Anxiety slope	Intercept (men)	Gender effect (W > M)	Men: anxiety slope	Women: anxiety slope	Slope interaction
Sensitivity	**β = 2.92**	β = 0.18	**β = 2.71**	β = 0.40	β = 0.06	β = 0.27	β = 0.21
	***p* < 0.01**	*p* = 0.17	***p* < 0.01**	*p* = 0.10	*p* = 0.76	*p* = 0.12	*p* = 0.39
Decision bias	β = −0.06	β = −0.04	β = −0.06	β = 0.10	β = −0.02	β = −0.06	β = −0.04
	*p* = 0.18	*p* = 0.24	*p* = 0.18	*p* = 0.12	*p* = 0.70	*p* = 0.17	*p* = 0.50
Metacognitive bias	**β = 6.48**	**β = −0.28**	**β = 6.47**	β = 0.02	**β = −0.46**	β = −0.13	β = 0.33
	***p* < 0.01**	***p* = 0.01**	***p* < 0.01**	*p* = 0.92	***p* < 0.01**	*p* = 0.37	*p* = 0.12
Insight	**β = −0.10**	**β = −0.09**	β = −0.07	β = −0.06	β = −0.07	**β = −0.11**	β = −0.04
	**HDI**	**HDI**	HDI	HDI	HDI	**HDI**	HDI
	**[−0.20, −0.01]**	**[−0.18, −0.01]**	[−0.20, 0.05]	[−0.23, 0.11]	[−0.19, 0.05]	**[−0.23, < 0.01]**	[−0.20, 0.12]

Interoceptive measure	Depression regression		Depression and gender regression				
	Intercept	Anxiety slope	Intercept (men)	Gender effect (W > M)	Men: anxiety slope	Women: anxiety slope	Slope interaction
Sensitivity	**β = 2.96**	β = 0.18	**β = 2.76**	β = 0.40	β = 0.29	β = 0.09	β = −0.20
	***p* < 0.01**	*p* = 0.17	***p* < 0.01**	*p* = 0.13	*p* = 0.17	*p* = 0.59	*p* = 0.47
Decision bias	β = −0.01	**β = −0.06**	β = −0.05	β = 0.09	β = −0.07	β = −0.06	β = 0.01
	*p* = 0.81	***p* = 0.04**	*p* = 0.24	*p* = 0.13	*p* = 0.13	*p* = 0.13	*p* = 0.84
Metacognitive bias	**β = 6.42**	**β = −0.32**	**β = 6.41**	β = 0.01	**β = −0.50**	β = −0.21	β = 0.29
	***p* < 0.01**	***p* < 0.01**	***p* < 0.01**	*p* = 0.95	***p* = 0.01**	*p* = 0.15	*p* = 0.21
Insight	**β = −0.15**	**β = −0.11**	β = −0.12	β = −0.06	β = −0.05	**β = −0.18**	β = −0.13
	**HDI**	**HDI**	HDI	HDI	HDI	**HDI**	HDI
	**[−0.25, −0.03]**	**[−0.21, −0.01]**	[−0.27, 0.01]	[−0.24, 0.13]	[−0.19, 0.08]	**[−0.34, −0.03]**	[−0.33, 0.07]

*Note:* Interoceptive sensitivity, decision bias and metacognitive bias analyses were performed using general linear models, with *p* < 0.05 denoting significance (two‐tailed). Interoceptive insight was analysed within the hierarchical HMeta‐d regression model against log (Mratio), with a 95% highest density interval of the posterior density that excludes zero denoting significance. Significant parameters are shaded grey.

The sensitivity analysis for state anxiety revealed that collapsing the study‐specific categorical variables rendered the main results for metacognitive bias and insight largely unchanged (Table 2). However, a qualitative difference was observed in the regression analyses for decision bias. Here, a significant negative relationship was revealed with state anxiety in the group as a whole (β = −0.06; *p* = 0.04) and in men alone (β = −0.10; *p* = 0.04) that was not previously present, with no significant gender interaction effect (women > men β = 0.06; *p* = 0.34) (Table 2).

**TABLE 2:** Sensitivity regression analyses for each interoceptive measure against state anxiety as a whole group (left columns) and accounting for gender (right columns), with no additional regressors for study site.

Interoceptive measure	State anxiety regression		State anxiety and gender regression				
	Intercept	Anxiety slope	Intercept (men)	Gender effect (W > M)	Men: anxiety slope	Women: anxiety slope	Slope interaction
Sensitivity	**β = 3.13**	β = −0.02	**β = 2.97**	β = 0.35	β = 0.18	β = −0.17	β = −0.35
	***p* < 0.01**	*p* = 0.86	***p* < 0.01**	*p* = 0.17	*p* = 0.38	*p* = 0.31	*p* = 0.19
Decision bias	β = 0.01	**β = −0.06**	β = −0.04	β = 0.10	**β = −0.10**	β = −0.04	β = 0.06
	*p* = 0.66	***p* = 0.04**	*p* = 0.35	*p* = 0.09	***p* = 0.04**	*p* = 0.27	*p* = 0.34
Metacognitive bias	**β = 6.40**	**β = −0.26**	**β = 6.39**	β = 0.02	β = −0.21	**β = −0.29**	β = −0.07
	***p* < 0.01**	***p* = 0.01**	***p* < 0.01**	*p* = 0.92	*p* = 0.20	***p* = 0.03**	*p* = 0.73
Insight	**β = −0.13**	**β = −0.08**	β = −0.09	β = −0.07	β = 0.03	**β = −0.17**	**β = −0.20**
	**HDI**	**HDI**	HDI	HDI	HDI	**HDI**	**HDI**
	**[−0.22, −0.05]**	**[−0.17, < 0.01]**	[−0.21, 0.02]	[−0.23, 0.09]	[−0.10, 0.16]	**[−0.30, −0.05]**	**[−0.38, −0.02]**

*Note:* Interoceptive sensitivity, decision bias and metacognitive bias analyses were performed using general linear models, with *p* < 0.05 denoting significance (two‐tailed). Interoceptive insight was analysed within the hierarchical HMeta‐d regression model against log (Mratio), with a 95% Highest Density Interval of the posterior density that excludes zero denoting significance. Significant parameters that were consistent with the original state anxiety analysis are shaded light grey, and significant parameters that were not previously significant in the original state anxiety analysis are shaded darker grey.

**FIGURE 3:**
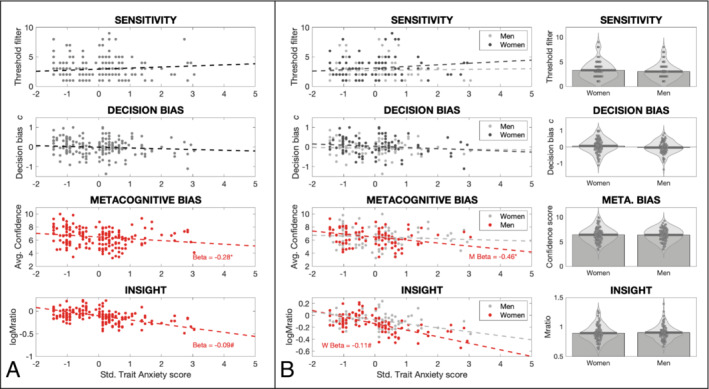
(A) Regression analyses of each of the four interoceptive measures against trait anxiety for the whole participant group. Both metacognitive bias and insight demonstrated a significant negative relationship with trait anxiety, with no effects observed for interoceptive sensitivity nor decision bias: Trait anxiety slope parameter for metacognitive bias, *p* = 0.01; trait anxiety slope parameter for metacognitive insight, HDI [−0.18, −0.01]. (B) Left: Regression analyses of each of the four interoceptive measures against trait anxiety for men and women separately: Trait anxiety slope parameter for metacognitive bias in men, *p* < 0.01; trait anxiety slope parameter for metacognitive insight in women, HDI [−0.31, −0.05]. Right: No mean differences were observed according to gender for any of the breathing interoceptive measures. *Significant result using ordinary least squares regression with *p* < 0.05. #Significant result using a hierarchical regression model with highest density interval excluding zero.

**FIGURE 4:**
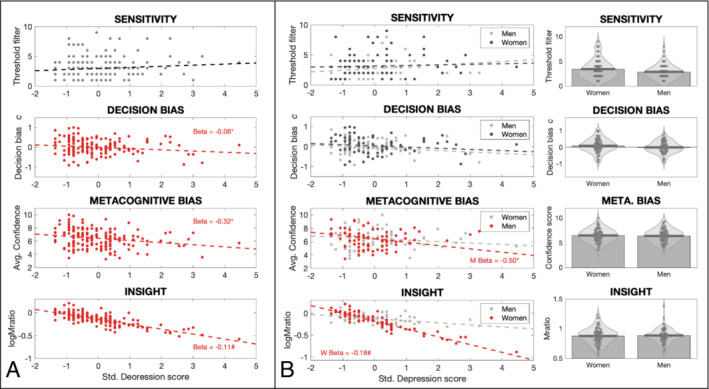
(A) Regression analyses of each of the four interoceptive measures against depression for the whole participant group. Both metacognitive bias and insight demonstrated a significant negative relationship with depression, with no effects observed for interoceptive sensitivity nor decision bias: Depression slope parameter for metacognitive bias, *p* = 0.01; depression slope parameter for metacognitive insight, HDI [−0.21, −0.01]. (B) Left: Regression analyses of each of the four interoceptive measures against depression for men and women separately: Depression slope parameter for metacognitive bias in men, *p* = 0.01; depression slope parameter for metacognitive insight in women, HDI [−0.34, −0.03]. Right: No mean differences were observed according to gender for any of the breathing interoceptive measures. *Significant result using ordinary least squares regression with *p* < 0.05. #Significant result using a hierarchical regression model with highest density interval excluding zero.

Finally, trait anxiety and depression also demonstrated a negative relationship with both metacognitive bias and insight across the whole group (Figures 3A and 4A, Table 1). For metacognitive bias there were no significant gender interaction effects in a similar manner to findings from state anxiety (trait anxiety women > men β = 0.33; *p* = 0.12: depression women > men β = 0.29; *p* = 0.21). For interoceptive insight, unlike for state anxiety, the gender interaction effect was not significant for either trait anxiety (women > men β = −0.04; HDI = [−0.20, 0.12]) or depression (women > men β = −0.13; HDI = [−0.33, 0.07]), despite consistently significant beta estimates in women (trait anxiety women β = −0.11; HDI = [−0.23, < 0.01]: depression women β = −0.18; HDI = [−0.34, −0.03]) (Figures 3B and 4B, Table 1).

## 4 Discussion

Consistent with previous work (Harrison, Köchli et al. 2021; Harrison, Marlow et al. 2021), we observed that elevated state anxiety was related to reduced overall confidence in interoceptive perceptions (‘metacognitive bias’) in all participants. However, state anxiety was only related to decreased interoceptive insight (congruency between confidence and correct/incorrect decisions) in women but not men (with a significant gender interaction effect), while on average performance across genders was similar for all interoceptive measures. These findings provide the first evidence that while absolute interoceptive abilities are not different between men and women, the relationship between state anxiety and interoceptive insight may differ with gender. Additionally, these findings may explain why this relationship between interoceptive insight and anxiety has previously been observed in study samples where the proportion of women was higher (Harrison, Marlow et al. 2021) compared to those with equal numbers of men and women (Harrison, Köchli et al. 2021). Finally, the previously reported finding of impaired interoceptive sensitivity with greater levels of anxiety (Harrison, Köchli et al. 2021) was not replicated here with a larger study sample, consistent with mixed findings previously reported for the respiratory domain (Harver, Katkin, and Bloch 1993; Prentice and Murphy 2022).

Interestingly, this gender‐based interaction between state anxiety and interoceptive insight did not translate to trait anxiety nor depression measures. Here, the relationship was still strongly significant for women, but a small negative association between insight and both trait anxiety and depression in men rendered the interaction effects insignificant. Furthermore, while not significantly different between genders, the relationship between metacognitive bias and both trait anxiety and depression were larger (and significant) in men but not women. Therefore, the findings from this study not only highlight differences in the relationship between anxiety and respiratory interoception across genders but also highlight differences between often highly correlated factors such as state anxiety, trait anxiety and depression.

Here, the study sample was maximised by pooling available data from across four study sites. While a total of 175 participants markedly improves statistical power beyond the analysis of each study separately, it is possible that some of the interactions and gender‐specific effects we failed to find might only become detectable in an even larger sample. Furthermore, additional heterogeneity may have been introduced via differences in study settings and protocols, and one could question whether our statistical model with site‐specific effects adequately captures this heterogeneity. However, a sensitivity analysis (without site‐specific effects) of the main study findings demonstrated only minor changes regarding the relationship between state anxiety, metacognitive bias and insight. In contrast, one qualitative difference was noted in this sensitivity analysis for decision bias, where a marginally significant relationship with state anxiety was revealed across both genders, and a significant relationship in men but not women (with no significant interaction effect). This relationship describes how greater levels of state anxiety relate to a greater likelihood of reporting the presence of an inspiratory resistance across all trials. Therefore, interoceptive variables such as decision bias may be more susceptible to the specifics of study settings and protocols, warranting further investigation as to its potential relationship with anxiety. A final limitation worth mentioning is that the cross‐sectional nature of the current study is unable to infer causality between anxiety, depression and different levels of interoceptive dysfunction. This limitation should be kept in mind when exploring clinical interventions that target interoceptive processes.

Despite these limitations, the current results may usefully inform the assessment and treatment of anxiety across genders. This hope is strengthened by the successful use of interoceptive interventions in other disorders. For example, novel cardiac interoceptive training strategies have recently been successfully employed to lower both state (Feinstein et al. 2018) and trait (Quadt et al. 2021) anxiety in adults with autism, with both strategies simultaneously demonstrating improvements in interoceptive qualities. However, while Quadt et al.'s (2021) training programme was able to improve cardiac‐related interoceptive sensitivity and metacognitive bias, interoceptive insight was not significantly enhanced across all participants. Importantly, gender differences were not considered either before or following interoceptive training, and therefore, it is possible that disparities between men and women may exist within these results provided the current findings apply across respiratory and cardiac modalities. While meta‐analytic findings focussed on interoceptive sensitivity have concluded that, on average, men are more accurate than women for cardiac interoceptive tasks, with mixed findings for respiratory tasks and no difference for gastric tasks (Prentice and Murphy 2022; Prentice et al. 2022), no studies have directly considered modality‐specific metacognitive measures, nor their gender‐specific relationship with anxiety and depression. Therefore, while the findings from the present study can only currently be considered regarding the inspiratory resistance domain, they suggest opportunities for devising and assessing new therapeutic intervention schemes in a gender‐dependent fashion, with the potential to address respiratory metacognitive biases for all anxious individuals, while specifically targeting respiratory interoceptive insight in anxious women.

## Author Contributions

**Olivia K. Harrison:** conceptualization, data curation, formal analysis, funding acquisition, investigation, methodology, project administration, visualization, writing – original draft. **Laura Köchli:** data curation, investigation, project administration. **Stephanie Marino:** data curation, investigation, project administration. **Lucy Marlow:** data curation, investigation, project administration. **Sarah L. Finnegan:** data curation, investigation, project administration. **Ben Ainsworth:** data curation, investigation, project administration. **Benjamin J. Talks:** data curation, investigation, project administration. **Bruce R. Russell:** project administration, supervision. **Samuel J. Harrison:** conceptualization, formal analysis, methodology, software, validation, visualization. **Kyle T. S. Pattinson:** data curation, funding acquisition, investigation, project administration. **Stephen M. Fleming:** conceptualization, investigation, methodology, software. **Klaas E. Stephan:** conceptualization, formal analysis, funding acquisition, investigation, methodology, project administration, resources, supervision, writing – original draft.

## Conflicts of Interest

The authors declare no conflicts of interest.

### Peer Review

The peer review history for this article is available at https://www.webofscience.com/api/gateway/wos/peer‐review/10.1111/ejn.16672.

## Data Availability

The data that support the findings of this study are available on request from the corresponding author. The data are not publicly available due to privacy or ethical restrictions. All code is available open access (https://github.com/IMAGEotago/BRIMA). All analyses were pre‐specified in a time‐stamped analysis plan (https://gitlab.ethz.ch/tnu/analysis‐plans/harrison_breathing_anxiety).
